# Supplementary material for: Lipid Profile Is Negatively Associated with Uremic Toxins in Patients with Kidney Failure—A Tri-National Cohort
Source: Toxins (Basel). 2022 Jun 16;14(6):412. doi: 10.3390/toxins14060412 (PMC9231137; doi:10.3390/toxins14060412)
Supplement: Supplementary file 1 [file toxins-14-00412-s001.zip › toxins-1712376-supplementary.pdf]

Supplementary Table S1: Univariate correlation analysis between uremic toxins and lipid profile in the entire cohort (N = 611)

**Analytes (µmol/l)**		**Total cholesterol (mmol/L)**	**HDL cholesterol (mmol/L)**	**Non-HDL cholesterol (mmol/L)**	**LDL cholesterol (mmol/L)**	**Remnant cholesterol (mmol/L)**	**TG (mmol/L)**
Indoxyl Sulphate	**r**	**-0.276**	**-0.232**	**-0.204**	**-0.227**	-0.008	-0.014
	**p**	**<0.001**	**<0.001**	**<0.001**	**<0.001**	0.853	0.744
p-Cresyl Sulphate	**r**	**-0.203**	-0.073	**-0.182**	**-0.164**	-0.099	-0.107
	**p**	**<0.001**	0.078	**<0.001**	**<0.001**	0.017	0.009
Indole-3 Acetic Acid	**r**	**-0.187**	-0.041	**-0.204**	**-0.200**	-0.119	-0.121
	**p**	**<0.001**	0.373	**<0.001**	**<0.001**	0.010	0.009
TMAO	**r**	**-0.259**	-0.078	**-0.257**	**-0.257**	-0.096	-0.098
	**p**	**<0.001**	0.059	**<0.001**	**<0.001**	0.020	0.017
Phenylacetylglutamine	**r**	**-0.299**	-0.129	**-0.268**	**-0.278**	-0.077	-0.082
	**p**	**<0.001**	0.002	**<0.001**	**<0.001**	0.062	0.048

Supplementary Table S1:

Univariate correlation analysis between uremic toxins and lipid profile in the entire cohort (N = 611). Exact Spearman's rank correlation coefficients, as well as their respective p values, for univariate correlations between the panel of five uremic toxins and six lipid parameters are depicted. Strict Bonferroni-correction was applied for all univariate analyses and Bonferroni-corrected level of significance was $p < 0.0016$ (0.05/30 tests). Abbreviations: HDL, High density lipoprotein; LDL, Low density lipoprotein; TG, Triglycerides; TMAO, Trimethylamine-N-Oxide. Significant associations ($p < 0.05$) are depicted in **bold**.

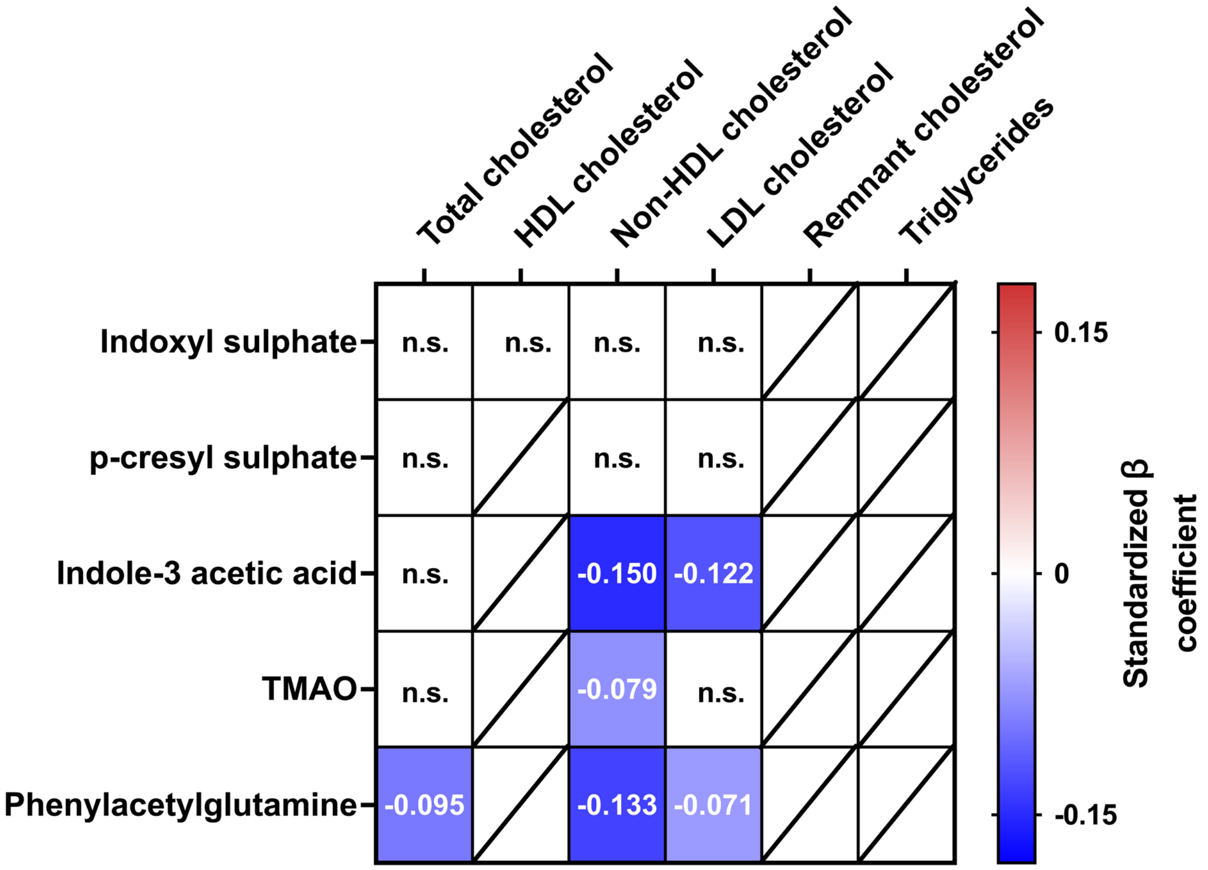

Supplementary Figure S1

Heatmap of standardized β coefficients for multiple linear regression analyses between the panel of five uremic toxins (dependent variable) and six lipid parameters adjusted for age, sex, study center, presence of diabetes, high sensitivity C-reactive protein (hsCRP), estimated glomerular filtration rate (eGFR), statin usage, dialysis treatment (yes/no), and body mass index (BMI) in the entire cohort (N = 611). A multivariate model was calculated only for those uremic toxins for which a Bonferroni-adjusted significant univariate correlation was found (Figure 1), otherwise the cell is crossed-out by a diagonal line. Non-normally distributed variables were log10 transformed prior to analysis. Non-significant multivariate models are indicated by n.s., whereas significant independent associations are depicted as color-coded cells with exact β coefficients inside. Thus, positive (in red/warmer colours) and negative associations (in blue/cooler colours) are shaded based on their respective standardized β coefficients. Abbreviations as indicated in Supplementary Table S1

Supplementary Table S2: Multivariate linear regression models for the associations between uremic toxins and lipid profile stratified by study center																			
**Analytes (µmol/l)**		**Stockholm subcohort (N = 235)**						**Leuven subcohort (N = 150)**						**Leipzig subcohort (N = 226)**					
		**TC (mM)**	**HDL-C (mM)**	**Non-HDL-C (mM)**	**LDL-C (mM)**	**Remnant C (mM)**	**TG (mM)**	**TC (mM)**	**HDL-C (mM)**	**Non-HDL-C (mM)**	**LDL-C (mM)**	**Remnant C (mM)**	**TG (mM)**	**TC (mM)**	**HDL-C (mM)**	**Non-HDL-C (mM)**	**LDL-C (mM)**	**Remnant C (mM)**	**TG (mM)**
Indoxyl sulphate	**β**	0.006	-0.089	0.068	0.041	-	-	-0.073	-0.080	-0.028	-0.006	-	-	-0.032	0.062	-0.048	-0.045	-	-
	**p**	0.916	0.168	0.272	0.510	-	-	0.551	0.525	0.819	0.958	-	-	0.409	0.114	0.201	0.234	-	-
p-cresyl sulphate	**β**	0.023	-	0.034	0.083	-	-	-0.177	-	-0.121	-0.056	-	-	0.097	-	0.075	0.122	-	-
	**p**	0.756	-	0.645	0.274	-	-	0.249	-	0.428	0.714	-	-	0.181	-	0.286	0.086	-	-
Indole-3 acetic acid	**β**	-0.067	-	**-0.254**	**-0.273**	-	-	-0.065		-0.166	-0.156	-	-	-0.058	-	-0.075	-0.024	-	-
	**p**	0.324	-	**0.023**	**0.017**	-	-	0.615		0.197	0.227	-	-	0.403	-	0.268	0.731	-	-
TMAO	**β**	-0.067	-	-0.093	-0.042	-	-	-0.065		-0.100	-0.078	-	-	-0.016	-	-0.029	-0.017	-	-
	**p**	0.324	-	0.167	0.547	-	-	0.615		0.436	0.543	-	-	0.744	-	0.554	0.729	-	-
Phenylacetylglutamine	**β**	-0.088	-	-0.016	-0.060	-	-	-0.125		-0.179	-0.066	-	-	**-0.076**	-	-0.085	**-0.079**	-	-
	**p**	0.150	-	0.817	0.335	-	-	0.200		0.187	0.496	-	-	**0.038**	-	0.076	**0.030**	-	-

Supplementary Table S2:

Multiple linear regression analyses stratified by the three different subcohorts from Stockholm (Sweden), Leuven (Belgium), and Leipzig (Germany) between five uremic toxins (dependent variable) and lipid parameters adjusted for age, sex, presence of diabetes, hsCRP, eGFR, statin usage, dialysis treatment (yes/no), and BMI. A multivariate model was calculated only for those uremic toxins for which a Bonferroni-adjusted significant univariate correlation was found (Figure 1). Non-normally distributed variables were log10 transformed prior to analysis. Standardized β coefficients, as well as the respective p-values, are given for each model. Significant associations ($p < 0.05$) after adjustment for covariates are depicted in **bold**. Abbreviations: C, Cholesterol; TC, Total cholesterol. All other abbreviations are indicated in Supplementary Table S1.

Supplementary Table S3: Multivariate linear regression models for the associations between uremic toxins and lipid profile stratified by dialysis treatment

Analytes (µmol/l)		Dialysis (N = 423)						Non-dialysis (N = 188)					
		TC (mM)	HDL-C (mM)	Non-HDL-C (mM)	LDL-C (mM)	Remnant C (mM)	TG (mM)	TC (mM)	HDL-C (mM)	Non-HDL-C (mM)	LDL-C (mM)	Remnant C (mM)	TG (mM)
Indoxyl sulphate	**β**	-0.009	-0.04	0.004	0.022	-	-	-0.051	-0.048	-0.033	-0.04	-	-
	**p**	0.862	0.436	0.941	0.661	-	-	0.305	0.264	0.486	0.415	-	-
p-cresyl sulphate	**β**	0.039	-	0.039	0.101	-	-	-0.077	-	-0.054	-0.058	-	-
	**p**	0.506	-	0.510	0.090	-	-	0.394	-	0.523	0.521	-	-
Indole-3 acetic acid	**β**	-0.039	-	**-0.137**	-0.106	-	-	**-0.323**	-	**-0.267**	**-0.233**	-	-
	**p**	0.547	-	**0.037**	0.111	-	-	**0.003**	-	**0.010**	**0.034**	-	-
TMAO	**β**	-0.035	-	-0.082	-0.033	-	-	-0.061	-	-0.066	-0.049	-	-
	**p**	0.502	-	0.119	0.533	-	-	0.362	-	0.297	0.462	-	-
Phenylacetylglutamine	**β**	**-0.132**	-	**-0.119**	**-0.093**	-	-	-0.027	-	-0.072	-0.032	-	-
	**p**	**0.003**	-	**0.044**	**0.041**	-	-	0.658	-	0.274	0.599	-	-

Supplementary Table S3:

Multiple linear regression analyses stratified by dialysis status between five uremic toxins (dependent variable) and lipid parameters adjusted for age, sex, presence of diabetes, hsCRP, eGFR, study center, statin usage, and BMI. A multivariate model was calculated only for those uremic toxins for which a Bonferroni-adjusted significant univariate correlation was found (Figure 1). Non-normally distributed variables were log10 transformed prior to analysis. Standardized β coefficients, as well as the respective p-values, are given for each model. Significant associations ($p < 0.05$) after adjustment for covariates are depicted in **bold**. Abbreviations are indicated in Supplementary Table S1 and S2.

Supplementary Table S4: Multivariate linear regression models for the associations between uremic toxins and lipid profile stratified by dialysis duration

Analytes (µmol/l)		Incident dialysis (N = 63)						Prevalent dialysis (N = 353)					
		TC (mM)	HDL-C (mM)	Non-HDL-C (mM)	LDL-C (mM)	Remnant C (mM)	TG (mM)	TC (mM)	HDL-C (mM)	Non-HDL-C (mM)	LDL-C (mM)	Remnant C (mM)	TG (mM)
Indoxyl sulphate	β	-0.202	-0.144	-0.040	-0.037	-	-	0.009	0.048	0.013	0.031	-	-
	p	0.177	0.367	0.789	0.802	-	-	0.867	0.392	0.806	0.576	-	-
p-cresyl sulphate	β	**0.460**	-	**0.385**	**0.454**	-	-	-0.017	-	0.018	0.050	-	-
	p	**0.002**	-	**0.008**	**0.002**	-	-	0.789	-	0.785	0.445	-	-
Indole-3 acetic acid	β	0.108	-	-0.138	-0.095	-	-	-0.065	-	**-0.147**	-0.115	-	-
	p	0.606	-	0.500	0.643	-	-	0.356	-	**0.039**	0.114	-	-
TMAO	β	**0.307**	-	0.075	0.183	-	-	-0.086	-	-0.105	-0.067	-	-
	p	**0.028**	-	0.595	0.191	-	-	0.131	-	0.068	0.253	-	-
Phenylacetylglutamine	β	**-0.279**	-	-0.025	**-0.257**	-	-	**-0.115**	-	**-0.133**	-0.072	-	-
	p	**0.018**	-	0.861	**0.027**	-	-	**0.018**	-	**0.042**	0.150	-	-

Supplementary Table S4:

Multiple linear regression analyses stratified by prevalent vs. incident dialysis status between five uremic toxins (dependent variable) and lipid parameters adjusted for age, sex, presence of diabetes, hsCRP, eGFR, study center, statin usage, and BMI. A multivariate model was calculated only for those uremic toxins and the respective lipid parameter for which a Bonferroni-adjusted significant univariate correlation was found (Figure 1). Non-normally distributed variables were log10 transformed prior to analysis. Standardized β coefficients, as well as the respective p-values, are given for each model. Significant associations ($p < 0.05$) after adjustment for covariates are depicted in **bold**. Abbreviations are indicated in Supplementary Table S1 and S2.

**Supplementary Table S5: Multivariate linear regression models for the associations between uremic toxins and lipid profile stratified by sex**														
**Analytes (µmol/l)**		**Male subjects (N = 399)**						**Female subjects (N = 212)**						
		**TC (mM)**	**HDL-C (mM)**	**Non-HDL-C (mM)**	**LDL-C (mM)**	**Remnant C (mM)**	**TG (mM)**	**TC (mM)**	**HDL-C (mM)**	**Non-HDL-C (mM)**	**LDL-C (mM)**	**Remnant C (mM)**	**TG (mM)**
Indoxyl sulphate	**β**	-0.072	**-0.082**	-0.025	-0.034	-	-	-0.002	0.079	-0.019	-0.001	-	-
	**p**	0.050	**0.019**	0.496	0.374	-	-	0.963	0.096	0.703	0.987	-	-
p-cresyl sulphate	**β**	-0.024	-	0.041	0.060	-	-	-0.005	-	0.056	0.007	-	-
	**p**	0.687	-	0.487	0.326	-	-	0.954	-	0.490	0.937	-	-
Indole-3 acetic acid	**β**	-0.061	-	-0.113	-0.120	-	-	-0.020	-	**-0.197**	-0.110	-	-
	**p**	0.335	-	0.085	0.077	-	-	0.163	-	**0.027**	0.229	-	-
TMAO	**β**	**-0.110**	-	**-0.118**	-0.091	-	-	0.032	-	0.-014	0.025	-	-
	**p**	**0.016**	-	**0.009**	0.051	-	-	0.597	-	0.818	0.689	-	-
Phenylacetylglutamine	**β**	**-0.091**	-	**-0.132**	-0.067	-	-	**-0.094**	-	**-0.129**	-0.077	-	-
	**p**	**0.013**	-	**0.008**	0.074	-	-	**0.037**	-	**0.039**	0.106	-	-

Supplementary Table S5:

Multiple linear regression analyses stratified by sex between five uremic toxins (dependent variable) and lipid parameters adjusted for age, presence of diabetes, hsCRP, eGFR, study center, statin usage, dialysis treatment (yes/no), and BMI. A multivariate model was calculated only for those uremic toxins and the respective lipid parameter for which a Bonferroni-adjusted significant univariate correlation was found (Figure 1). Non-normally distributed variables were log10 transformed prior to analysis. Standardized β coefficients, as well as the respective p-values, are given for each model. Significant associations ($p<0.05$) after adjustment for covariates are depicted in **bold**. Abbreviations are indicated in Supplementary Table S1 and S2.
